# Deciphering the potential efficacy of acetyl-L-carnitine (ALCAR) in maintaining connexin-mediated lenticular homeostasis

**Published:** 2012-07-25

**Authors:** Arumugam Ramachandran Muralidharan, George Leema, Thangaraj Annadurai, Thirugnanasambandhar Sivasubramanian Anitha, Philip A. Thomas, Pitchairaj Geraldine

**Affiliations:** 1 Department of Animal Science, School of Life Sciences, Bharathidasan University, Tiruchirappalli – 620024, Tamilnadu, India; 2 Institute of Ophthalmology, Joseph Eye Hospital, Tiruchirappalli – 620001, Tamilnadu, India

## Abstract

**Purpose:**

To determine the putative role of acetyl-L-carnitine (ALCAR) in maintaining normal intercellular communication in the lens through connexin.

**Methods:**

In the present study, Wistar rat pups were divided into 3 groups of eight each. On postpartum day ten, Group I rat pups received an intraperitoneal injection (50 µl) of 0.89% saline. Rats in Groups II and III received a subcutaneous injection (50 µl) of sodium selenite (19 µmol/kg bodyweight); Group III rat pups also received an intraperitoneal injection of ALCAR (200 mg/kg bodyweight) once daily on postpartum days 9–14. Both eyes of each pup were examined from day 16 up to postpartum day 30. Alterations in the mean activity of the channel pumps, calcium-ATPase and sodium/potassium-ATPase, were determined. The expression of genes encoding key lenticular gap junctions (connexin 46 and connexin 50) and a channel pump (plasma membrane Ca^2+^-ATPase [*PMCA1*]) was evaluated by reverse transcription-PCR. Immunoblot analysis was also performed to confirm the differential expression of key lenticular connexin proteins. In addition, bioinformatics analysis was performed to determine the interacting residues of the connexin proteins with ALCAR.

**Results:**

Significantly lower mean activities of Ca^2+^-ATPase and Na^+^/K^+^ -ATPase were observed in the lenses of Group II rats than those in Group I rat lenses. However, the observed mean activities of Ca^2+^-ATPase and Na^+^/K^+^-ATPase in Group III rat lenses were significantly higher than those in Group II rat lenses. The mean mRNA transcript levels of the connexin 46 and connexin 50 genes were significantly lower, while the mean levels of *PMCA1* gene transcripts were significantly higher, in Group II rat lenses than in Group I rat lenses. Immunoblot analysis also confirmed the altered expression of connexin proteins in lysates of whole lenses of Group II rats. However, the expression of connexin 46 and connexin 50 proteins in lenses from group III rats was essentially similar to that noted in lenses from normal (Group I) rats. Hydrogen bond-interaction between ALCAR and amino acid residues at the functional domain regions of connexin 46 and connexin 50 proteins was also demonstrated through bioinformatics tools.

**Conclusions:**

The results suggest that ALCAR plays a key role in maintaining lenticular homeostasis by promoting gap junctional intercellular communication.

## Introduction

Cataract, characterized by loss of lenticular transparency, is the most common cause of preventable blindness worldwide [1,2]. At present, the most effective treatment of cataract is the surgical removal of the opacified lens. However, the cost of surgery may place it beyond the reach of economically-deprived individuals [3]; moreover, since the surgery need to be done, ideally, by an ophthalmologist, there is often a huge backlog in the number of patients awaiting surgery. Thus, preventing or delaying the progression of cataract formation by pharmacological means may alleviate these problems to a great extent.

Abnormally elevated lenticular levels of sodium and calcium, and a decreased level of potassium, have been reported in senile cortical cataracts [4]. Intracellular free calcium has long been recognized as an important regulator of cellular events through an array of calcium-mobilizing receptors and signaling proteins [5]. Influx of calcium into the lens, leading to activation of calpain, a cysteine proteinase enzyme responsible for disintegration of crystallin protein, has been well documented in cataract animal models [6-9]. Na^+^/Ca^2+^ exchanger, plasma membrane Ca^2+^-ATPase (PMCA) and sarcoplasmic/endoplasmic reticular calcium-ATPase are the three crucial membrane-bound transport proteins that are reported to regulate transport of Ca^2+^ and Na^+^ ions. Hence, defects in expression, localization or function of ion transport proteins and gap junction proteins have been implicated in the formation of cataract [10]. So also, Li et al. [11] reported that maintenance of lenticular homeostasis and transparency depends on an extensive network of gap junctions. Connexins (connexin 43, connexin 46, and connexin 50) constitute the major component of lenticular gap junctions [12].

Antioxidants have generally been considered to protect against oxidative stress-induced cellular damage, but the effects of individual antioxidants may differ, depending on their structure and the dosage at which they are effective [13]. Acetyl-L-carnitine (ALCAR) is a naturally-occurring quaternary amine, synthesized endogenously in the human brain, liver, and kidneys by the acetyl carnitine transferase enzyme or obtained from dietary sources [14]. We have previously demonstrated that ALCAR acts as an effective antioxidant and, possibly, as an inhibitor of calpain activity in preventing selenite-induced cataractogenesis [8,15], however, its influence on maintaining lenticular homeostasis by mediating membrane transporters has not been explored. In the present study, the potential efficacy of ALCAR in maintaining lenticular homeostasis by virtue of maintaining lenticular connexin proteins was evaluated experimentally in the lenses of Wistar rats; an attempt was also made to employ bioinformatics tools to determine which, if any, residues of the connexin proteins interact with the drug, ALCAR.

## Methods

### Experimental animals

Nine day-old rat pups (Wistar strain) were used in this study. The pups were housed with parents in large spacious cages, and the parents were given food and water ad libitum. These animals were used in accordance with Institutional guidelines and with the Association for Research in Vision and Ophthalmology Statement for the Use of Animals in Research. The rat pups were randomly divided into three groups of eight each, Group I, which received only saline (normal); Group II, which received selenite alone (selenite-challenged, untreated) and Group III, which received selenite and ALCAR (selenite-challenged, ALCAR-treated).

Each rat pup in groups II and III received a single subcutaneous injection (50 µl) of sodium selenite (19 µmol/kg bodyweight) on postpartum day 10. In addition, pups in group III received intraperitoneal injections (200 mg/kg bodyweight) of ALCAR (Sigma–Aldrich, St. Louis, MO); the first dose of ALCAR was administered 1 day before the selenite injection and was repeated once daily for five consecutive days thereafter. On postpartum day 16, the animals were sacrificed and the lenses were removed.

### Preparation of supernatant of lens for biochemical analysis

Prior to biochemical analysis, the lenses dissected out by the posterior approach from each group were washed with normal physiologic saline, weighed, and processed. Each lens was homogenized in 10 times its mass of 50 mM phosphate buffer (pH 7.2) and centrifuged at 17,000× g for 15 min at 4 ^◦^C. The supernatant obtained was used for analysis of ATPase activity. Protein concentration in each sample was estimated by the method of Bradford [16], using BSA as a standard.

### Quantitative analysis of activities of ATPases

The activity of Ca^2+^-ATPase was determined by the method of [17] and the activity of Na^+^/K^+^-ATPase was determined essentially as described by [18]. The activities of Ca^2+^-ATPase and Na^+^/K^+^-ATPase were measured by evaluating the inorganic phosphorous released by splitting of ATP molecules into ADP by ATPases in the presence of Ca^2+^ ions and Na^+^/K^+^ ions, respectively. Inorganic phosphate reacts with ammonium molybdate to form phosphomolybdate. The hexavalent molybdenum of phosphomolybdate is reduced by 1-amino 2-napthol 4-sulphonic acid (ANSA) to give a blue-colored complex, which is measured at 620nm. Enzyme-specific activity was expressed as nmol Pi released per min per mg of protein.

### Reverse transcription (RT)-PCR analysis of mRNA transcript level

Lenses for this experiment, after removal from rat eyes, were immediately homogenized in Trizol (Sigma-Aldrich) reagent (1 ml/100 mg tissue) and RNA was isolated per the manufacturer’s protocol. RNA’s were quantified using a UV-spectrophotometer at 260 nm and RNA integrity was checked via agarose gel electrophoresis by assessing 18S and 28S band intensities. RT–PCR was performed using a one-step RT–PCR kit (Qiagen, Hilden, Germany), per the manufacturer’s instructions, with the gene-specific primers for connexin 46, connexin 50, and plasma membrane calcium ATPase 1 (*PMCA1*), and for β-actin (*ACTB*; the ‘housekeeping’ gene; Table 1). Amplification was done in an Eppendorf thermal cycler (Hamburg, Germany).

**Table 1:** Primer sequences and the expected product sizes of the genes amplified.

**S. No.**	**Genes**	**Primer sequences 5′-3′**	**PCR product size (bp)**
1.	connexin 46	Forward primer 5′-CATTCTGGTGCTGGGGGCGG-3′	290
		Reverse primer 5′-CGGCCGTGCTGAGGGTTGTC-3′	
2.	connexin 50	Forward primer 5′-GTCTCGCAGCAACGGGGGTG-3′	209
		Reverse primer 5′-GGGGCAGGATGCGGAAACCA-3′	
3.	PMCA1	Forward primer 5′-GACTCGCCACTGAAGGCGGT-3′	454
		Reverse primer 5′-GGCTTCCCGCCAAACTGCAC-3′	
4.	β-actin	Forward primer 5′-ATCGCTGACAGGATGCAGAAG-3′	107
		Reverse primer 5′-AGAGCCACCAATCCACACAGA-3′	

Abbreviations: PMCA1; Plasma Membrane Calcium ATPase 1.

After completion of the PCR reaction, 10 μl of each PCR product were analyzed by gel electrophoresis on a 2% agarose gel. The ethidium bromide-stained gel was subjected to densitometric scanning and the band intensity of the cDNA fragment of the genes was normalized against the band intensity of cDNA fragment of the internal control (*ACTB*) gene, using Quantity One Software (Bio Rad, Hercules, CA).

### Immunoblotting

The lenses were homogenized in sample buffer containing 62.5 mM Tris–HCl (pH 6.8), 2% sodium dodecyl sulfate (SDS), 5% β-mercaptoethanol, protease inhibitor cocktail (1:100) and 2 mM PMSF. After homogenization, lysates were sonicated on ice and centrifuged at 17,000× g for 15 min at 4 °C following the method of [19]. The supernatant obtained was used for immunoblot analysis. Proteins subjected to sodium dodecyl sulfate PAGE (SDS–PAGE) were electrophoretically transferred to a polyvinylidene fluoride (PVDF) membrane using a semidry blotting apparatus (Bio-Rad). Blotting was done at 25 V for 1 h. Blotted membranes were stained by Ponceau S solution to check for the efficiency of transfer; subsequently, blocking was done with 5% non-fat milk powder in Tris buffer saline (pH 7.5) with 0.1% (v/v) Tween-20 for 3 h, following the method of Towbin et al. [20]. Specific antibodies against connexin 46 (Santa Cruz Biotechnology, Inc., Santa Cruz, CA), connexin 50 (a kind gift from Dr. Linda Musil, Oregon Health and Science University, Portland, OR), and β-actin (Sigma) were used. Immunoreactivity was visualized with horseradish peroxide conjugated to anti-goat IgG secondary antibody and hydrogen peroxide/4-chloro 1-napthol.

### Bioinformatics analysis

Homology modeling of the functional domains of connexin 46 and connexin 50 was performed using Modeler 9v7 software [21]. The amino acid sequences of connexin 46 and connexin 50, corresponding to *Rattus norvegicus,* were retrieved from GenBank (P29414.2 and NP_703195.2, respectively) in National Center for Biotechnology Information (NCBI). The domain region of connexin 46 and connexin 50 were scanned using the Prosite database [22]. The domain was then subjected to Position-Specific Iterative BLAST (PSI-BLAST) search [23] against Protein Data Bank (PDB) [24]. Templates having a sequence identity >35% were selected and aligned using Clustal W [25]. Subsequently, homology modeling was performed for the domain regions of connexin 46 and connexin 50, employing the appropriate template. The model was validated using SAVES server. To gain insight into the putative binding mode of connexin 46 and connexin 50 domains with the drug, ALCAR, molecular docking analysis was performed using AUTODOCK 4 [26]. A semi-flexible docking protocol was employed by keeping target protein domains rigid while the ligand being docked was kept flexible.

In addition to the modeling approach, the prediction of possible kinase-specific phosphorylation sites of the connexin 46 and connexin 50 proteins of *Rattus norvegicus* was performed using NetPhosK 1.0 server [27] with a threshold of 0.6.

### Statistical analysis

Statistical analysis was performed with Statistical Package for Social Sciences (SPSS) software package for Windows (Version 16.0; IBM Corporation, Armonk, NY). Differences between all experimental groups were assessed by one-way ANOVA. Post-hoc testing was performed for intergroup comparisons using the least significance difference test. The experiments were performed at least three times with duplicate samples. P-values <0.05 were considered statistically significant.

## Results

### Quantitative analysis of ATPase activity

In the present study, the mean activities of Ca^2+^-ATPase and Na^+^/K^+^-ATPase were found to be significantly lower in the lenses of selenite-challenged, untreated (Group II) rats than those in normal (Group I) rat lenses (Table 2). However, the observed mean activities of Ca^2+^-ATPase and Na^+^/K^+^-ATPase in Group III (selenite-challenged, ALCAR-treated) rat lenses were significantly higher than the values in Group II rat lenses (although still lower than the values in Group I lenses).

**Table 2:** Quantitative analysis of lenticular ATPase activity in Wistar rat lenses.

**ATPase activity (µmoles of Pi liberated/min/mg protein)**	**Group I rat lenses**	**Group II rat lenses**	**Group III rat lenses**
Ca^2+^ ATPase*	15.041±0.35	10.825±0.61^a,b^	13.184±0.41^a^
Na^+^/K+ ATPase*	13.847±0.56	12.619±0.40^a,b^	13.59±0.34^a^

*All values are expressed as mean±SD of five determination. Group I: Normal. Group II: Selenite-challenged, untreated. Group III: Selenite-challenged, acetyl-L-carnitine treated. ^a^ Value significantly different (p<0.05) from Group I value. ^b^ Value significantly different (p<0.05) from Group III value.

### Effect of ALCAR on mRNA transcript levels

**Figure 1:**
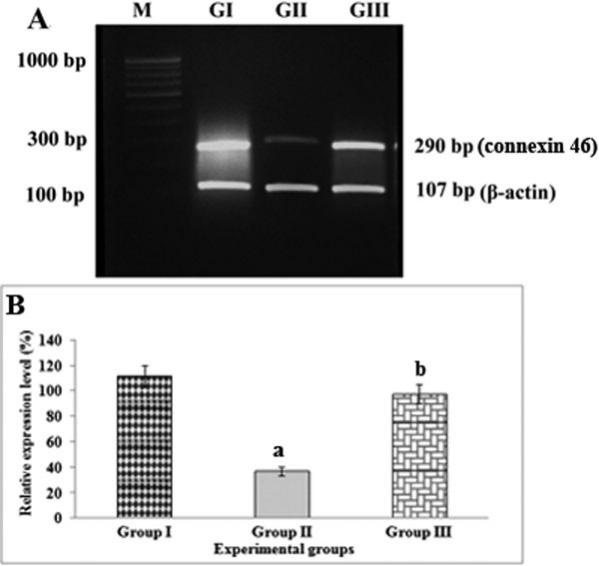
Semi-quantitative reverse transcription-PCR analysis of connexin 46 mRNA in rat lenses visualized on an ethidium bromide-stained agarose gel alongside mRNA of  β-actin (*ACTB*). **A**: M- 100 bp DNA ladder; GI- Group I (Normal); GII- Group II (Selenite-challenged, cataract-untreated); GIII- Group III (Selenite-challenged, acetyl-L-carnitine-treated). **B**: The results depicted are normalized to levels of β-actin gene. Data are mean value  (experiments run in triplicate) of ratios of intensity for gene of interest divided by that for β-actin. ^a^Group I versus Group II & III values (p<0.05); ^b^Group II versus Group III values (p<0.05).

**Figure 2:**
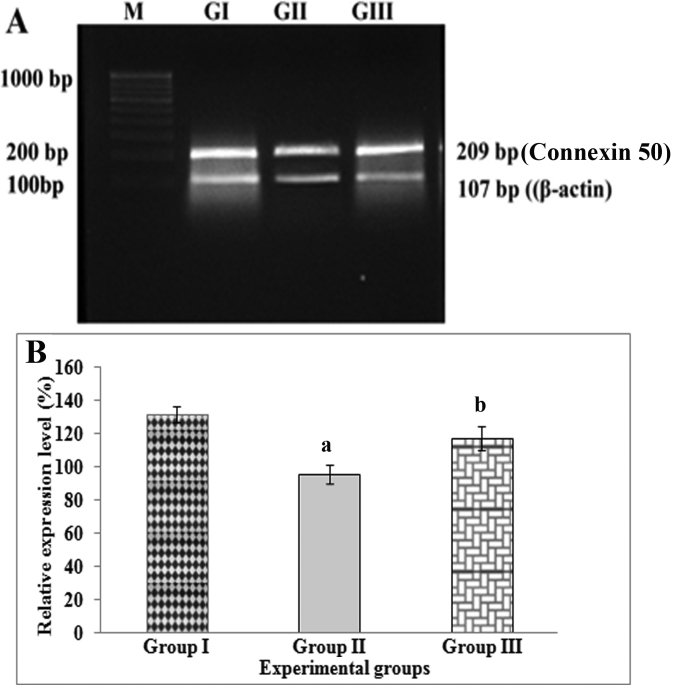
Semi-quantitative reverse transcription -PCR analysis of connexin 50 mRNA in rat lenses visualized on an ethidium bromide-stained agarose gel alongside mRNA of β-actin (*ACTB*). **A**: M- 100 bp DNA ladder; GI- Group I (Normal); GII- Group II (Selenite-challenged, cataract-untreated); GIII- Group III (Selenite-challenged, acetyl-L-carnitine-treated). **B**: The results depicted are normalized to levels of *ACTB*. Data are mean value (experiments run in triplicate) of ratios of intensity for gene of interest divided by that for *ACTB*. ^a^Group I versus Group II & III values (p<0.05); ^b^Group II versus Group III values (p<0.05).

**Figure 3:**
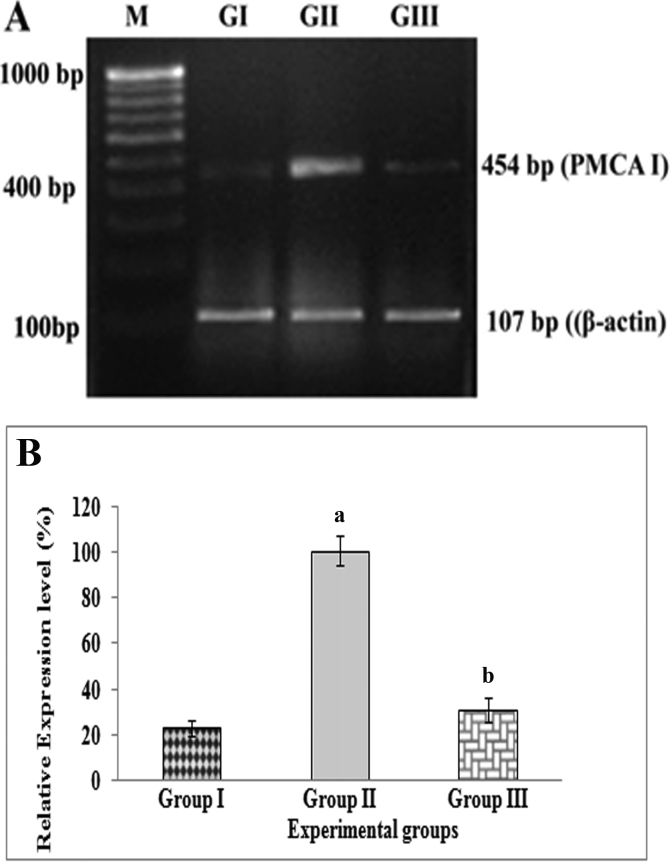
Semi-quantitative reverse transcription -PCR analysis of plasma membrane calcium ATPase 1 (*PMCA1*) mRNA in rat lenses visualized on an ethidium bromide-stained agarose gel alongside mRNA of β-actin (*ACTB*). **A**: M- 100 bp DNA ladder; GI- Group I (Normal); GII- Group II (Selenite-challenged, cataract-untreated); GIII- Group III (Selenite-challenged, acetyl-L-carnitine -treated). **B**: The results depicted are normalized to levels of *ACTB*. Data are mean value (experiments run in triplicate) of ratios of intensity for gene of interest divided by that for *ACTB*. ^a^Group I versus Group II & III values (p<0.05); ^b^Group II versus Group III values (p<0.05).

In lenses of Group II rats (selenite-challenged, untreated), the mean levels of connexin 46 (Figure 1) and connexin 50 (Figure 2) gene transcripts were found to be significantly (p<0.05) lower while the mean level of the *PMCA1* (Figure 3) gene transcript was found to be significantly (p<0.01) higher than those in lenses of Group I (normal) rats. In lenses of Group III rats (selenite-challenged, ALCAR-treated), the mean levels of the transcripts of connexin 46 and connexin 50 were significantly higher while that of *PMCA1* was significantly lower, when compared to the values in Group II rat lenses, and in fact, were thus found to approach normal levels.

### Immunoblot analysis of connexin 46 and connexin 50

**Figure 4:**
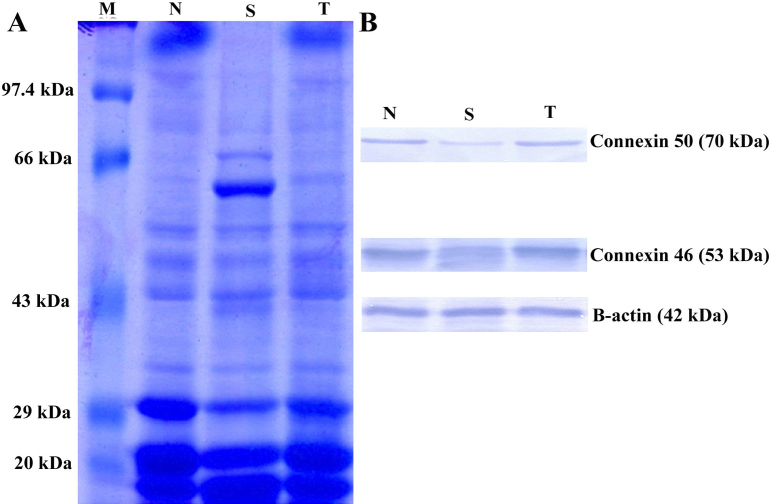
Sodium dodecyl sulfate PAGE (SDS-PAGE) and immunoblot analysis of connexin 46 and connexin 50 in lysate of whole lens. **A**: Electrophoretic pattern of proteins stained with Coomassie Brilliant Blue. **B**: Representative western blots for connexin 46 and connexin 50 (with β-actin as loading control) in lenses of 16-day-old Wistar rat pups. M-Marker; N- Group I (normal); S- Group II (selenite-challenged, cataract untreated); T- Group III (selenite-challenged, ALCAR-treated).

To further validate the data on mRNA transcript levels of connexin 46 and connexin 50, immunoblot analysis was performed with specific antibodies against connexin 46 and connexin 50. In lenses of 16-day-old normal (Group I) rats, connexin 46 and connexin 50 bands were found to be of high intensity, whereas in lenses of selenite-challenged, untreated (Group II) rats, the bands of both proteins were found to be of very low intensity. However, in selenite-challenged, ALCAR-treated (Group III) rat lenses, the intensity of the bands corresponding to connexin 46 and connexin 50 was greater than that noted in Group II rat lenses. The protein loading control β-actin was confirmed by the specific antibody (Figure 4).

### Bioinformatics analysis

**Figure 5:**
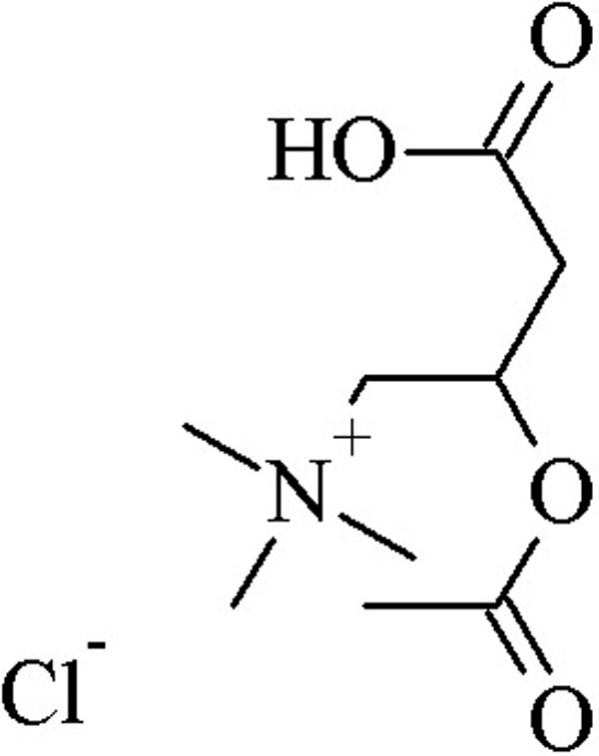
2-dimensional chemical structure of acetyl-L-carnitine (ALCAR).

**Figure 6:**
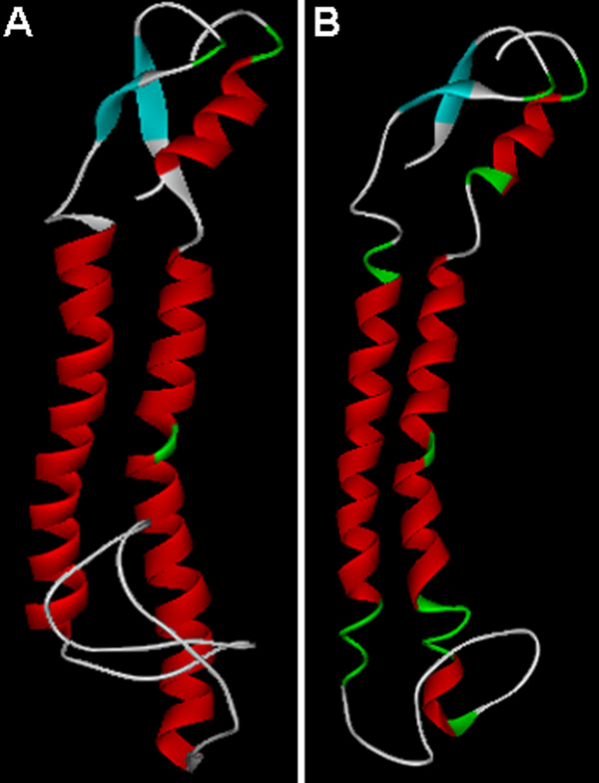
Modeled structure of the connexin46 and connexin 50 domains obtained from Modeler 9v7. The structure is displayed in secondary structure mode using Pymol. **A**: Homology model of connexin 46 domain. **B**: Homology model of connexin 50 domain.

**Figure 7:**
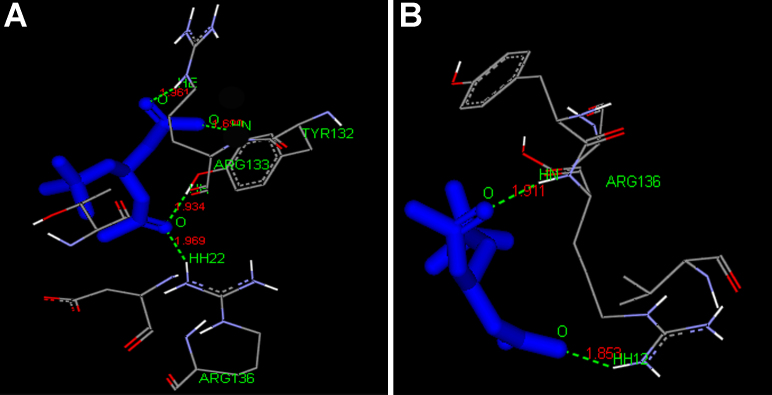
Binding pose of the best conformation obtained for connexin 46 (Cx46) and connexin 50 (Cx50) domains with acetyl-L-carnitine (ALCAR). **A**: Cx 46 binding pose. **B**: Cx 50 binding pose. The proposed binding modes of the drug molecule are shown in a ball and stick model. Hydrogen bonds are shown as *dotted green lines*, and the bond length between the interacting amino acid residues of the domain and the drug molecule, ALCAR, is indicated. The ARG 133, ARG 136 and TYR 132 residues that interact with ALCAR are also represented.

To explore the interaction or binding of ALCAR (Figure 5) with domains of connexin 46 and connexin 50, we docked ALCAR into the binding pocket of modeled connexin 46 and connexin 50 domains (Figure 6). As can be seen from Figure 7, hydrogen bond interaction appears between ALCAR and amino acid residues TYR 132, ARG 133, and ARG 136, of connexin 46 and ARG 136 of connexin 50, which are all located at the functional domain regions of the connexin 46 and connexin 50 proteins.

The NetPhosK prediction results revealed that many of the serine and threonine residues were predicted to be phosphorylated by several kinases, both in connexin 46 and connexin 50. Six and twelve protein kinase C (PKC) phosphorylation sites were predicted on connexin 46 and connexin 50, respectively (Table 3 and Table 4).

**Table 3:** Predicted protein kinase C (PKC) phosphorylation sites within the aminoacid sequence of connexin 46 of *Rattus norvegicus*.

**Phosphorylation sites**	**Scores**
THR 19	0.71
THR 24	0.62
SER 402	0.64
SER 405	0.75
SER 406	0.90
SER 438	0.64

**Table 4:** Predicted protein kinase C (PKC) phosphorylation sites within the aminoacid sequence of connexin 50 of *Rattus norvegicus*.

**Phosphorylation sites**	**Scores**
THR 27	0.63
SER 134	0.85
SER 138	0.82
SER 139	0.74
SER 140	0.61
SER 141	0.90
SER 142	0.86
THR 145	0.91
SER 241	0.73
SER 266	0.78
SER 300	0.74
SER 430	0.87

## Discussion

The lens, with its unique structural and physiologic properties, is an organ that is vulnerable to oxidative stress and related complications. Maintenance of the balance between active transport and passive leakage of ions is believed to regulate the cytosolic concentrations of sodium, potassium and calcium in lenticular cells [10]. Biswas et al. [28] have suggested that unregulated Ca^2+^-mediated proteolysis of essential lenticular proteins by calpains is a major factor contributing to the formation of cataract, both in animals and humans. Various ion channels, gap junctions and passive transporters are employed in the regulation of intra-lenticular concentrations of ions, while any abnormality in the expression, localization, or function of ion transport proteins has been reported to contribute to the formation of cataract [10]. The disruption in calcium homeostasis appears to be an important component in the cascade of events leading to cataract development in animal models [8,29].

In selenite cataractogenesis, oxidative stress-induced lipid peroxidative damage of lenticular membranes contributing to the loss of Ca^2+^ATPase activity and subsequent accumulation of Ca^2+^ has been reported by Shearer et al. [7]. Thus, the activity of lenticular Ca^2+^-ATPase and Na^+^/ K^+^-ATPase possibly depends on the lipid composition of the membrane [30]. Oxidative stress has been found to inhibit Na^+^/K^+^ pumps, followed by an uncoupling of ATP hydrolysis and ion translocation, leading to lenticular opacification [31,32]. Antioxidant therapy has been reported to prevent elevation of calcium levels in the selenite-challenged lens, thereby inhibiting the activation of calpain [29,33]. So also, the antioxidant, ALCAR, has been reported to prevent elevations in lenticular calcium levels in selenite-challenged rats [8].

In the present study, the mean activities of Ca^2+^ATPase and Na^+^/K^+^ATPase were found to be significantly lower in Group II rat lenses (exposed to selenite alone) than the mean activities in Group I rat lenses (normal). This possibly occurred due to the oxidation of critical sulfhydryl groups, leading to the inactivation of Ca^2+^-ATPase and Na^+^/K^+^-ATPase, a phenomenon that has been reported previously by Borchman et al. [34] and Shearer et al. [7]. Babizhayev et al. [35] reported that lipid peroxidation also causes oxidative inhibition of Ca^2+^ATPase in the lens. However, in the present investigation, such a decline in mean activities of these ATPases was prevented in lenses of rats that had been treated with ALCAR (Group III). Hence, the higher (compared to Group II) mean activities (approximating normal values) of Ca^2+^-ATPase and Na^+^/K^+^-ATPase in Group III lenses suggests that ALCAR protects against oxidation of sulfhydryl groups in the lenticular epithelium, thereby maintaining the activity of these ATPases at near normal levels. Previous studies on the activity of Ca^2+^-ATPase in untreated cataractous lenses and cataractous lenses treated with flavonoids isolated from *Vitex negundo* and broccoli have reported similar effects [29,33]. The data on Na^+^/K^+^-ATPase activity are consistent with those obtained by Graw [36,37] and Hightower et al. [38], who have reported that increased Na^+^/K^+^-ATPase activity is associated with cataract development.

Cellular calcium homeostasis is achieved by a balance between inward leak and an outward ‘push’ of calcium by plasma membrane calcium-ATPase (PMCA) and Na^+^/Ca^2+^ exchanger (NCX). Internal sequestration of calcium relies on sarcoplasmic/endoplasmic reticular calcium-ATPase to shift calcium into the endoplasmic reticulum which functions as an intracellular calcium store [10]. PMCA utilizes energy to pump Ca^2+^ ions out of the cytosol into the extracellular milieu, usually against a strong chemical gradient [39]. In addition to PMCA, connexin 46 and connexin 50 play a significant role in maintaining lenticular homeostasis, and are reported to prevent cataract formation [11,40]. Hence, in the present investigation, an attempt was made to assess the mRNA transcript levels of genes encoding connexin 46, connexin 50 and *PMCA1* in the lenses of the experimental groups of rats. The mean transcript levels of connexin 46 and connexin 50 were found to be significantly lower, while the mean transcript level of PMCA1 was found to be significantly higher, in lenses of selenite-challenged, untreated (Group II) rats, when compared to those of normal (Group I) rat lenses. The mean transcript levels of connexin 46, connexin 50, and *PMCA1* genes in selenite- challenged, ALCAR-treated (Group III) rat lenses, were found to be at near normal levels. The downregulation of connexin 46 and connexin 50 gene expression in lenses of Group II rats was possibly due to alterations to post-transcriptional mechanisms [41-43] which subsequently caused disruption of the gap junctions. However, relatively increased expression levels in Group III lenses, when compared to the levels in Group II lenses, suggests a possible role for ALCAR in preventing such alterations to post-transcriptional mechanisms and disruption of the gap junctions. The upregulation of *PMCA1* gene expression in group II lenses suggests that there was a breakdown in lenticular homeostatic regulation, leading to increased lenticular calcium levels. Similar observations on upregulation of PMCA1, as well as increased lenticular calcium levels, have been reported in human lenticular epithelial cells after exposure to H_2_O_2_ and thapsigargin [44] and also in the UPL hereditary cataract model [45]. The maintenance of near normal mean transcript levels of PMCA1 in lenses of Group III rats (selenite-challenged, ALCAR-treated) suggests a modulatory effect for ALCAR in maintaining lenticular calcium homeostasis.

Rodent lens fiber cells express the gap junction proteins connexin 46 and connexin 50. Connexin 43 is present only in the epithelial layer of the lenticular tissue, whereas connexin 46 and connexin 50 are predominant in the cortical fiber cells of the lens [46]. According to White [47] connexin 46 and connexin 50 expressed in fiber cells exhibit different roles in normal lenticular growth and in maintenance of lenticular transparency. Hence, in the present study, we have focused on the modifications occurring in connexin 46 and connexin 50 alone. Fleschner [48] showed that a disruption of the functioning of gap junctions may be an important factor in the mechanism of cataract formation induced by sodium selenite. Tang et al. [49] reported that nuclear cataract formation was associated with the loss of connexin 46, following which there was a massive elevation of lenticular calcium levels, resulting in over-activation of the calpain isoforms. In the present investigation, the data on mRNA transcript levels of connexin 46 and connexin 50 were validated on performing immunoblot analysis. It was found that the relative expression of both the proteins was significantly less in group II rat lenses than that in group I rat lenses. Since connexins are phosphoproteins [50,51] the phosphorylation of connexins by protein kinase C (PKC), especially PKCγ, plays a crucial role in the disruption of gap junctions, thus leading to the loss of intercellular communication [19,52]. Lampe and Lau [53] also suggested that lenticular gap junctions tend to change communication properties in a manner that is correlated with changes in phosphorylation of connexin. Recently, Shearer et al. [54] reported phosphorylation of serine residues in both the cytoplasmic loop and COOH-terminus region of connexin 50. In the present study, such a decline of both connexin 46 and connexin 50 appeared to have been prevented in ALCAR-treated (Group III) rat lenses. A possible explanation for the modulation of connexin 46 and connexin 50 in Group III rat lenses is that ALCAR prevented alterations at both post-transcriptional and post-translational levels, thereby enhancing intercellular communication at the gap junctions; however, this hypothesis warrants further investigation. The results of the present investigation support the notion that prevention of nuclear cataract formation requires an array of metabolites transported through gap junctions, to prevent aberrant changes in lenticular proteins and to maintain the lenticular homeostasis needed for transparency of the lens [55].

In the present study, homology modeling of connexin 46 and connexin 50 functional domains and subsequent docking with ALCAR was performed to gain insight into the most probable binding conformation of the domain region of the connexin protein with the drug, ALCAR. Our docking studies suggest that the affinity of ALCAR with domains of connexin 46 and connexin 50 occurs due to hydrogen bonding between the receptor and the drug molecule, the bond length being <3 Å, which indicates a strong interaction between the ligand and the domain region. The strong hydrogen bond interaction between the ligand, ALCAR and the domain regions of connexin 46 and connexin 50 is mainly due to the constituent atoms involved in the atomic interaction. The oxygen belonging to ester group of ALCAR acts as an hydrogen bond acceptor whereas the hydroxyl group of TYR132, and gunidino group (NH_2_) of ARG 133 and ARG 136, corresponding to the connexin 46 domain, acts as an hydrogen bond donor. Similarly, the oxygen belonging to carboxylate group of ALCAR acts as an hydrogen bond acceptor whereas the gunidino group (Nε) and the main-chain nitrogen of ARG 136, corresponding to the connexin 50 domain, acts as an hydrogen bond donor and thereby forms an hydrogen bond between the protein and ligand. Hence, it may be hypothesized that the interaction between the domain regions of connexin 46 and connexin 50 with ALCAR promotes the lenticular homeostasis that is mediated by the gap junction proteins. In future, it may be possible to modify the functional groups of the ALCAR molecule so that the analog of ALCAR, possessing the effective functional group, is given, it yields results that are as good as those obtained when the native ALCAR compound is administered. In the present investigation, an attempt was also made to predict the possible PKC phosphorylation sites. The results generated by this analysis suggested that SER 406 residue of connexin 46 and THR 145 and SER 430 residues of connexin 50 were the PKC phosphorylation sites predicted to possess high scores. PKC phosphorylation on these residues possibly altered the expression of connexin 46 and connexin 50 in selenite-challenged rat lenses.

In conclusion, the results generated through these experiments suggest that ALCAR upregulates the expression of the connexin proteins connexin 46 and connexin 50 and downregulates *PMCA1* gene expression, thereby preventing elevation of lenticular calcium levels and subsequent aggregation of crystallins in the lens. The observations made in the present study thus suggest the potential role of ALCAR in maintaining connexin-mediated lenticular homeostasis, and therein preventing selenite cataractogenesis.
